# Reasons given by pregnant women for participating in a clinical trial aimed at preventing premature delivery: a qualitative analysis

**DOI:** 10.1186/s12884-019-2240-8

**Published:** 2019-03-20

**Authors:** Thaís M. Monteiro, Leila Katz, Silvana F. Bento, Melania M. Amorim, Patrícia C. Moriel, Rodolfo C. Pacagnella

**Affiliations:** 1 Instituto de Medicina Integral Prof. Fernando Figueira (IMIP), Recife, Pernambuco Brazil; 2 Prof. Dr. José A Pinotti Women’s Hospital, Center of Integral Services for the Health of Women (CAISM), University of Campinas (UNICAMP), Campinas, SP Brazil; 3 School of Medical Sciences, University of Campinas (UNICAMP), Campinas, SP Brazil

**Keywords:** Pregnancy, Premature delivery, Clinical trial

## Abstract

**Background:**

In clinical trials, pregnant women are potentially vulnerable, and the fetus is exposed to the intervention. This study aimed to identify the reasons that led pregnant women at a high risk of premature delivery to participate in a randomized clinical trial.

**Methods:**

The women participating in the main trial were contacted by telephone postpartum and invited to answer an open questionnaire in a cross-sectional study. Data were collected by telephone and analyzed using thematic analysis. After the analysis categories were defined, all the answers were reviewed, categorized and grouped. A descriptive summary of the content of each category was then made.

**Results:**

Overall, 208 women from different geographical regions of the country agreed to participate. Four categories were identified: 1) The risk of losing the baby; 2) A previous experience of premature delivery; 3) The role of the doctor and other health professionals, and 4) The availability of quality medical care and free medication. The main reason given for agreeing to participate was to reduce the risks associated with the baby being born prematurely, particularly when the woman herself or someone close to her had already experienced premature delivery. Other reasons were having received clear guidance and explanations from the doctor regarding prematurity and about the study and being given the opportunity to receive free treatment with greater access to the public healthcare system.

**Conclusions:**

The decision to participate in a clinical trial is not easy, particularly when the individual is vulnerable and in a critical situation as in the case of a pregnant woman at a high risk of delivering prematurely. Fears and uncertainties regarding the pregnancy outcome, as well as the woman’s previous experiences and her awareness of the actual risks she faces will affect her decision regarding whether or not to participate. Recruitment challenges could be overcome by ensuring that the research team provides adequate information and support, thus creating a bond with participants that would foster a sense of safety and trust in the study proposals.

**Electronic supplementary material:**

The online version of this article (10.1186/s12884-019-2240-8) contains supplementary material, which is available to authorized users.

## Background

Clinical trials are intended to generate medical and scientific knowledge, contributing to the development of more effective and at times more innovative treatments. The willingness of patients to participate in a given clinical trial is a sine qua non for that trial to be conducted. However, it is exceedingly challenging to recruit pregnant women as study participants, and the gaps in existing knowledge on this group of women may serve as motivation for them to participate in research [1, 2].

Pregnancy is considered a normal physiological state; nevertheless, most women will use at least one pharmaceutical drug during pregnancy that they did not use previously, in addition to the vitamins and mineral supplements required during that time [3]. In the United States, approximately two out of every three pregnant women are prescribed medicines during pregnancy. Prescribing medication during pregnancy is often based on limited scientific evidence insofar as the drug’s safety and effectiveness are concerned [4]. Indeed, because most studies are conducted with non-pregnant women, the data generated may not apply in full to pregnant women [5].

Up to the 1990s, the United States Food and Drug Administration (FDA) recommended that pregnant women should be excluded from clinical trials for their own protection, the intention being to protect the fetus from the risks involved in participating in scientific research [6]. At the same time, this attempt to protect the fetus was offset by the risk of submitting pregnant women to treatments approved in studies conducted exclusively with non-pregnant women [5, 7].

Randomized clinical trials conducted in critical situations such as pregnancy are necessary in order to establish the safety of the product under evaluation and to supply scientific evidence of the outcome [8]. Pregnant women have traditionally been recognized as a potentially vulnerable population [9, 10]; therefore, it is particularly important that their experiences and perspectives, especially in critical situations, are understood [11, 12].

Currently, the FDA permits and even encourages the responsible inclusion of pregnant women in clinical trials as long as patient monitoring and safety is assured [13]. Nevertheless, there are still some limitations with respect to randomized clinical trials involving pregnant women, since these women are in a situation of vulnerability and because the fetus will also receive the proposed intervention [14].

The experience of women regarding the use of pharmaceutical drugs during pregnancy and their adherence to drug therapy are subjects that have seldom been evaluated. In general, pregnant women tend not to comply with drug therapy and/or to submit themselves to interventions during pregnancy through fear that they will lose their baby and/or that the treatment could cause undesirable effects on the fetus, even when the proposed treatment has been shown to cause no harm [3]. Therefore, when invited to participate in a clinical trial, the woman may withhold consent if she believes that participation could represent a risk to the baby or, conversely, she may feel pressured to agree to interventions that could benefit the fetus [14]. This decision is made even more difficult when the study involves high-risk pregnancies.

Various factors may interfere in the pregnant woman’s decision to participate or not in scientific research. The objective of the present study was to determine the reasons given by pregnant women for participating in a clinical trial and submitting themselves to interventions during pregnancy.

## Methods

This was a cross-sectional study based on the qualitative analysis of open questions used to evaluate women’s experience of participating in the *Pessary Plus Progesterone to Prevent Preterm Birth (P5) Study* (Trial registration RBR-3t8prz).

### The P5 study

Briefly, the P5 randomized controlled trial involves women at a high risk of preterm labor and has been ongoing since July 2015 in 17 Brazilian centers. The principal objective is to compare the efficacy of progesterone alone versus progesterone associated with cervical pessaries for the prevention of preterm birth in pregnant women with a short cervix. The study also aims to evaluate whether its results will justify universal cervical length screening and determine a treatment policy for the Brazilian population.

Pregnant women with a short uterine cervix, i.e. cervical length ≤ 30 mm as assessed by transvaginal ultrasonography screening at 18 to 22 weeks of pregnancy, are invited to participate in the clinical trial in which they may receive 200 mg of vaginal micronized progesterone alone or progesterone associated with a cervical pessary. Following randomization, women are followed up until 10 weeks after the infant’s birth to evaluate neonatal outcomes.

During recruitment to the clinical trial, patients are given an explanation on the risk of prematurity and the P5 study protocol. The woman should clearly understand that participating in the study does not guarantee a better gestational outcome, that she is free to decide whether to participate, and that her decision will not affect the treatment she and her child would receive at the healthcare unit. The patients are provided with explanations regarding the most common side effects of using vaginal progesterone (constipation, nausea, vaginal discharge) and the cervical pessary (intense vaginal discharge, leucorrhea, vaginal pain, cervical necrosis and painful intercourse). The information provided orally to the patient and on the informed consent form is believed to be sufficient to allow the women to reach a decision regarding their participation in the study.

### The cross-sectional qualitative study

The present cross-sectional study was part of a strategy within the P5 Study to identify barriers to developing a treatment to prevent preterm birth. The main objective was to obtain information on the experience of pregnant women regarding medical management for the prevention of preterm birth. This study also aimed to provide further clarification regarding the barriers that exist within the recruitment process and during the follow-up of critically ill and vulnerable patients in a clinical trial. For this purpose, a structured questionnaire containing both open and closed questions was administered to women who had completed the follow-up period of the P5 study, which extended until July 2017, meaning that all the women were interviewed 10 weeks after delivery.

Eligible women were contacted by telephone and invited to participate in the cross-sectional study. For those who did agree to participate, an appointment was made at a convenient date and time for each woman. Since the interviews were conducted by telephone, informed consent was given verbally, and the women’s agreement to participate in the study was recorded. The informed consent form was read to the women invited to participate in the study and they were given the opportunity to ask questions and clarify any doubts. When the women were satisfied with the information and with the answers they had received, they were asked if they would agree to participate in the study and their consent was recorded.

The internal review boards of the University of Campinas (UNICAMP) and the *Instituto de Medicina Integral Professor Fernando Figueira* (IMIP) approved the study protocol under reference numbers CAEE 5592.3016.1.0000.5404 and CAEE 3841.7114.0.2005.5201, respectively.

The 36-item questionnaire used in the cross-sectional study included 8 open questions about the women’s experience of using a treatment to prevent prematurity (Additional file 1). These questions were related to women’s understanding of the doctor’s explanations, prematurity, risk perception, experiences of her participation in the study, doubts about whether it was appropriate for the woman to participate in the study and her perception regarding the success of the treatment provided. The questions were asked directly and clearly.

The interviews were conducted in the Portuguese language and lasted on average around 10 minutes. The women’s answers were recorded and registered in a digital version of the questionnaire, with the responses to the open questions being transcribed *ipsis litteris* into the database. The interviews were conducted between January and July 2017 by investigators with experience in this interview technique. For this article, which reports on the reasons given by women for agreeing to participate in a clinical trial, a qualitative analysis was performed of two open questions directly linked to our objectives: 1) What made you finally decide to undergo the proposed treatment? and 2) In your opinion, did the treatment work or not? Why?

The participants’ responses were analyzed until saturation was reached. Data analysis was conducted according to the guidelines proposed by Patton [15]. The text corresponding to the two questions was read several times by two of the investigators to identify the units of analysis in the women’s answers that would meet the proposed objectives. Based on the defined set of units of analysis, categories of analysis were then proposed. After the categories had been defined, all the answers were read again, categorized and grouped together. A descriptive summary of the content of each category was then made.

This paper presents the results obtained for the following categories of analysis:

- The risk of losing the baby;
- A previous experience of premature delivery;
- The role of the doctor and other health professionals;
- Quality medical care and free medication

## Results

A total of 356 women were eligible for inclusion in the study. Of these, 127 could not be contacted by telephone and 21 refused to participate. Most of the women who refused to participate gave no reason for doing so. Those who did provide an explanation stated that the outcome of their pregnancy had been negative.

Interviewers stopped asking the open questions when the analysis reached saturation, which occurred at around 200 answers. Overall, 208 women with a mean age of 27.8 years were interviewed. Of these, 63.1% reported having completed high school, while 19.4% had completed elementary school, 15.0% had graduated from university and 2.4% had completed postgraduate studies. Self-reported ethnicity revealed that 39.8% of the women considered themselves white, 9.2% black and 50.5% brown. The majority (79.3%) stated that they lived with a partner. In addition, 22.8% of the women had already been affected by prematurity and 94.1% had had a singleton pregnancy.

### The risk of losing the baby

The main reason given by the women for agreeing to participate in the study was their concern about the risk of losing the baby. Their worry regarding the baby’s life was expressed at various moments throughout the interviews, with many stating explicitly that they were afraid of losing their baby, that participating in the study was “*the only way to save the baby*”, “*the only way out*”, … “*my last chance*”. They mentioned that they would do anything available to prevent premature delivery and by doing so prolong the pregnancy and “*save the baby*”.

“I did it to try to keep the child, right. To avoid the risk of having her too early or maybe even of losing her before that” (Example 117699).

The fear and anxiety of experiencing a high-risk pregnancy was a strong influence in the woman’s decision to participate in the study. For some, this fear was triggered by a previously unknown condition identified and explained by the healthcare professional involved in the study. Learning of the study’s existence and, consequently, of the treatment aimed at avoiding premature delivery offered the women new hope of being able to prolong their pregnancy for as long as possible. For some women, agreeing to participate meant protecting the baby, making the pregnancy safer and thus increasing the likelihood of a good outcome despite the fact that the effects of the treatment were still unknown.

“Actually…when I discovered that I was pregnant…. I had undergone insemination. I lost one baby at three months but the other one went on; so it was a much loved and longed for child. I would never have given up. I would have gone after a solution even if it were in another state, another country, but I didn’t want to lose my child” (Example 10).

One patient commented that she was afraid that if she didn’t agree to participate in the study her baby would be born prematurely. The role the mother assumes as guardian of her fetus’s well-being is clear from this comment, as is the responsibility she undertakes with respect to the health of the fetus - agreeing to participate in scientific research because she is afraid that refusing to do so would place her baby’s health at risk.

“*Because I was afraid of not doing it and losing the baby*” (Example 117857).

Some of the women agreed to participate in the study because they had other conditions that affected their risk of premature delivery in addition to their diagnosis of a short cervix, further increasing their likelihood of prematurity.

“My cervix was short, but not that short. It was 29 [millimeters]…but I also had another risk factor because I had a bicornuate uterus and I thought: “if I have a risk factor and I can benefit from something that is going to prevent my baby being born early, then I’m going to agree to it” so I decided to accept” (Example 119573).

### A previous experience of premature delivery

For some women, previous negative experiences of premature delivery and/or gestational losses of their own or of someone close to them made them fearful of experiencing another similar situation and this was the principal motivating factor behind their decision to agree to the treatment. The women expressed concern about having a premature child because survival is low, because the baby could suffer various health problems and might have to be admitted to an intensive care unit (ICU). Some women mentioned that prematurity could lead to complications for the babies over the short- and long-term, requiring extensive periods in incubators in the neonatal ICU, and that this was their reason for participating in the study. Even women who had not experienced this personally but who were close to people who had gone through such an experience were also affected by it.

“The other babies, it was hard having to see them in the ICU, when the only thing I could do was watch over them. So, I really wanted to have a pregnancy that would go to term, to be able to leave the delivery room with my baby. For that reason, I would have done just about anything that they said would help, no matter what” (Example 46).

“I decided first of all because I had my first pregnancy and I lost a baby…in my second pregnancy, when I knew I was pregnant again, my second baby was born at 32 weeks, premature too, and he stayed in the ICU for a month. When he (the doctor) told me that there was this medicine to hold in the premature baby, I decided to go for it. I knew there was a likelihood that this third child of mine would be born prematurely too, that it wouldn’t go the whole nine months. So, with this medicine my baby went up to 36 weeks” (Example 36).

### The role of the doctor and other health professionals

The explanation given by the doctors regarding the diagnosis of short cervix and the proposed treatment influenced the women’s decision to participate in the study. Since they were fragile/vulnerable at that moment because of what they were going through, the role of the doctor was crucial in the process of deciding whether to participate in the study.

According to the women, the information provided by the doctors was clear, comprehensive and given in such a way that they were able to understand what was being explained. The information provided included the explanation that this was a research study and that agreeing to participate was no guarantee that the pregnancy would successfully go to term. The women quoted the doctors as saying that: “*it was not definite that it* [the treatment] *would actually work”* and that… “*it might not be possible to maintain the pregnancy*”.

The conversations between the doctors and the women established a bond of trust between them. This made the women feel more secure with respect to the problem they were facing and the treatment that was being proposed, thus enabling them to reach a conscious decision regarding whether or not to participate in the study.

“In fact, the doctor’s explanation was very important for me. He discussed all the possibilities with me and made me feel safe…” (Example 37).

“Because in actual fact there was no conclusive evidence that the treatment would actually work, but I agreed to do it because if by doing so there was a chance that my baby would be born at term, I wanted to take advantage of it irrespective of anything else” (Example 25).

### Referral made by the doctor providing prenatal care

In some cases, it was the doctors providing prenatal care who referred the women to the centers in which the P5 study was being conducted. These women mentioned that their doctors considered the treatment to be safe and effective for avoiding premature delivery. In addition, certain women were referred because they had a history of a previous high-risk pregnancy. Referral by the doctor providing prenatal care made these women feel safer and more confident about agreeing to participate in the study. When given a diagnosis of short cervix and when the risk of premature delivery was explained to them, some women, who had not been referred by their gynecologists, called their doctors to ask their opinion on whether or not to participate in the study.

“I realized that it would be better to ensure that my pregnancy went to term and also because my doctor had said that it is a very safe and effective method to avoid the risk of premature delivery” (Example 1).

### Quality medical care and free medication

Some women praised the care they received during their participation in the study. They mentioned that it was quality care provided by attentive professionals, who gave them the information they needed in such a way that it was easily understood. They were also given reassurance about the tests to be carried out and also about the place in which the study was being conducted. In addition, they received the treatment free of charge. All these actions carried out by the research team contributed towards reassuring the woman, both with respect to her own participation and that of her child. The possibility of easy access to treatment and follow-up in the study center was also mentioned. Only one patient considered that the information regarding the high risk of prematurity given by the healthcare professional following the diagnosis of a short cervix was not given in a humanized manner. She was very upset when leaving the hospital and had no accompanying person with her at the time. Nevertheless, she underwent the treatment and considered it valid.

“Right from the very first consultation, they were very clear with me and gave me lots of attention. So, I felt that this was additional care that I was getting for my child. They provided attention and monitoring that we really need, irrespective of whether we have private healthcare insurance or if we depend on the National Health Service (SUS). The assurance that they gave me was substantial, right from the very first day” (Example 116345).

“For me it was marvelous [having participated in the study]. I had no doubts whatsoever and I am just grateful that I was given the opportunity to use the pessary and progesterone” (Example 18).

Several of the women stated that one of the reasons for having agreed to participate in the study was the fact that it was free.

“It is a very [expensive] treatment…the devices, the medication that is provided and everything. It’s very expensive. So, it’s free healthcare, isn’t it? Both benefit from it, right? It benefits those who are doing all this, right, and I benefit from the study too”. (Example 116345).

Many of the women understood that the pessary was offered as an alternative to cervical cerclage since it was already too late to perform cerclage at that more advanced stage of their pregnancy. Those women agreed to participate in the study because “*it was the only option available to me*”. One patient reported a previous negative experience with cervical cerclage and for that reason she decided to participate in the study.

“Because that was the most viable option”. (Investigator: Most viable for what?). “To avoid the risk of premature delivery and, since I was already at an advanced stage, I couldn’t have cerclage anymore so this was my only option” (Example 115301).

“Because I had undergone cervical cerclage previously and it was very painful. So, I opted to insert the pessary because I thought it would be better…. I thought that with the pessary I would be able to move around easier, walk about. With my uterus sewn up, it would be very painful for me” (Example 116005).

A few patients agreed to participate in the study to be able to contribute to the development of scientific research and help other mothers avoid premature delivery.

“Firstly, as a precaution; it would help avoid premature delivery, but also to help your study get answers, bearing in mind that this process is still under evaluation. So, to be able to make my contribution too and also to be monitored by a professional” (Example 113499).

## Discussion

For the great majority of the pregnant women participating in this study, the main reason for agreeing to participate in the P5 clinical trial was their fear of losing the pregnancy. This fear was based not only on their current situation but also on the women’s perceived risk based on a previous experience of premature delivery, either their own experience or that of someone close to them. The information and instructions given by the health professional, together with the trust they had in this professional, were also mentioned as reasons behind the women’s decision to participate in the study, as well as the opportunity to obtain easier access to healthcare services.

For these women, participating in the study offered them the possibility of protecting their babies. Faced with a situation that could expose the pregnancy to considerable risks, these women agreed to participate in the study in the hope of improving their child’s chances despite being told by the study team that this was a research study and that there was no guarantee that the treatment would improve the perinatal outcome. However, it is true that a pregnant woman experiencing a critical situation and in a condition of vulnerability places her hope and faith in the health professionals and the treatment offered by them. Some women, therefore, may have felt compelled to participate in the study because of fears or doubts that not accepting the treatment would result in harm to their child. Even so, other women who were identified at ultrasound screening as being at a high risk of prematurity chose not to participate in the clinical trial because they did not believe that the risk justified exposing themselves and their baby to an experimental treatment. Likewise, a qualitative study reported that the principal motivation given by pregnant women for having participated in a scientific study was the possibility that it would benefit their baby to some extent. Even knowing that it was an experimental study, in general the mothers concluded that participating would cause no harm and that this would, therefore, be the best decision [8].

Previous studies, conducted to evaluate the motives that lead pregnant women to withhold their consent to participate in clinical trials, showed that the risk of exposing themselves and their fetus to a treatment that is still undergoing evaluation is deemed unacceptable to the women and represents a significant barrier to recruitment [16, 17]. In the present study, few women expressed fear or misgivings regarding use of the cervical pessary, with this seldom being given as a reason for not participating in the study. Since the benefits of this treatment in preventing premature delivery have yet to be confirmed and since the pessary is inserted into the pregnant woman’s vagina, women may find it difficult to understand both the mechanism of action and the pessary location. Such uncertainties may have weighed heavily in their decision not to participate in the study. None of the women reported fear or misgivings regarding the use of progesterone. This could be explained by the fact that progesterone is already available on the market as a treatment for premature delivery, and many women have already used it or know other women who have done so; hence, it is already well known to the patient as an established treatment.

Many women agreed to participate in the clinical trial after talking to the healthcare professional. Based on the information given to these women by the medical team during screening and when their cervix was being measured, they became aware of their increased risk of prematurity and the complications that could develop as the result of premature delivery. The clear and detailed way in which the information was provided, inspiring confidence and a sense of security in the women and establishing a good doctor-patient relationship, was mentioned several times by the women as being important factors when deciding whether or not to participate in the study. This shows the importance of the doctor-patient relationship in the decision-making process, since the pregnant woman begins to gain confidence in what is being proposed. In addition, the pregnant woman also believes that participating in the study will not result in any negative consequences, since the health professional would clearly not expose her to any risk. Similar findings were reported in a qualitative study that evaluated 22 pregnant women in premature labor participating in a clinical trial [8].

A multicenter study involving 3733 women found that the principal motivating factor in the decision to participate in a clinical trial was how well the study was explained, while the risk of unknown side effects was the principal barrier [17]. Similar findings were reported from a qualitative study involving 296 pregnant women who had refused to participate in a clinical trial involving the use of dietary supplements. The conclusions of that study were that the role of the research team is fundamental in fostering the woman’s confidence in the study by ensuring that she clearly understands the risks and benefits associated with her participation and allowing her to feel listened to and supported. Implementing such actions would increase participant recruitment [16]. Furthermore, a good doctor-patient relationship creates a bond between the two that will last throughout the entire study, increasing not only recruitment but also compliance with the study follow-up. This relationship between the P5 healthcare professionals and the women represents a possible a source of bias and may have influenced the patient’s perception of risk and the need for treatment. The fact that some women who refused to participate in this cross-sectional study stated that the outcome of their pregnancy had been negative might suggest the presence of bias, since more women whose pregnancy outcomes were negative may have refused to participate. This may constitute a limitation of the present study.

According to some patients, the fact that the doctor providing their prenatal care, who was not part of the P5 study team, had given them information and recommended the treatment was their reason for agreeing to participate in the study, reinforcing the influence of the doctor’s opinion in the decision-making process. A qualitative study that evaluated 22 patients participating in the ORACLE 1 and 2 clinical trials involving pregnant women with a risk of infection found that the principal factor influencing the woman’s decision to participate in the study concerned the socio-emotional aspects involved in the relationship between the health professional and the patient [8]. The women considered that the critical nature of the situation hampered their ability to absorb the information provided; nevertheless, they viewed the information provided by the professional in writing or verbally in a positive and favorable manner [18].

Therefore, the manner in which the information on the clinical trial is presented to the woman, including the language and terminology used, can affect her understanding of the study [19], influencing her decision to participate in the scientific research or not [20, 21]. In addition, it affects her satisfaction during the study [22] and increases patients’ perception of the competence of the professionals [23]. This strong relationship found between the pregnant woman and her obstetrician/gynecologist has already been reported in a study conducted to compare factors affecting the decision of the emergency patient regarding whether or not to participate in clinical trials in different areas of health. In that study, a good doctor-patient relationship was considered one of the main factors motivating the women to participate in research in the field of obstetrics/gynecology, whereas one of the major barriers was lack of trust in the doctor. This association was not found to the same degree in other medical specialties [24]. In the present study, the close bonding between the women and the healthcare providers was obvious from the statements made by most of the patients, and this could constitute a limitation of the study. Unfortunately, a bias such as this is a common occurrence since the care and the attention that clinical trial participants usually receive is somewhat different.

For patients with a prior experience of prematurity, either a personal experience or that of someone close to them, the decision regarding whether or not to participate in the study was strongly affected by this past experience. Experiencing prematurity and the consequences associated with it such as the baby having to be admitted to an ICU, sequelae in the newborn infant, and in the most severe cases, the baby’s death, so marked these women that when receiving a diagnosis of short cervix and being informed of their increased risk of prematurity, these memories returned and were decisive in their agreement to participate in the study. This process was associated with parents’ psychological disorders and traumas, since the parents were unprepared to deal with the stress caused by the child’s clinical condition and the need for intensive care [25]. When the outcome of their experience with prematurity was poor, the women were clearly even more eager to participate in the study, since in this study having experienced a previous negative outcome generated even more fear of losing another baby.

In the present study, the partner’s opinion was not mentioned as a factor influencing the woman’s decision regarding whether or not to participate in the study. The women decided by themselves whether to participate in the study or not, and this highlights their autonomy and self-sufficiency in choosing what they believed to be correct for them and for their child when in a situation of vulnerability such as pregnancy. In addition, the women did not mention that participating in the study would mean having access to a treatment for the prevention of premature delivery (cervical pessary) that was not yet available within the Brazilian National Health Service. Conversely, results of another study showed that the pregnant women who agreed to participate in a clinical trial did so because it appeared beneficial and the treatment offered was not available to anyone outside the study [26].

Some of the women stated that being able to contribute to science and help other women have their children was yet another reason for them to agree to take part in the study; however, it was not the principal motivating factor. This has been mentioned in other studies, but in general it is associated with the belief that it is acceptable to do so in benefit of others as long as it involves no risk or harm to the participant or her child [8, 16, 24].

Some of the women mentioned that gaining better access to healthcare services weighed strongly in their decision to participate in the study. One study involving pregnant women also found that women who agreed to take part in a clinical trial tended to believe that they would receive better care and have greater access to health professionals and to the medical tests they would require during pregnancy [8, 27, 28]. In developing countries such as Brazil, where access to public healthcare is often difficult and limited, participating in research could provide a woman with treatment that she would not ordinarily have. These women are able to access medical consultations, tests and treatments much faster than they normally would. In the case of the participants in the P5 study, the women had greater access to ultrasonography and more frequent medical consultations, including easier access to doctors, who even gave the women their cellphone number so that they would be contactable at any time to answer any questions. All this may also have influenced the women’s satisfaction with the treatment and strengthened the doctor-patient bond.

Interestingly, few patients refused to participate in the present study. As these patients had been monitored practically throughout their entire pregnancy and also following childbirth, they could have been expected to refuse to participate in yet one more stage, including interviews, particularly since another study reported that extended patient follow-up proved to represent a barrier to participation in scientific research [17]. This result is probably related to the manner in which the entire recruitment process and follow-up was presented and offered to the patient in the P5 study, providing comfort and a sense of safety to such a degree that the women were happy to be able to contribute in other stages of the study.

One of the strongpoints of the study refers to the substantial number of participants, which allowed adequate categories of analysis to be established and the principal barriers and reasons affecting the women’s decision to participate in the study to emerge. Furthermore, evaluating what leads pregnant women in developing countries, where education in health and knowledge regarding clinical trials are limited, to participate in studies is interesting and will certainly serve to guide the planning of future projects.

In addition to the limitations already mentioned, there is the fact that, although the women confirmed having understood the information provided regarding the design of the clinical trial and the entire protocol and follow-up, this was not checked. The degree to which the candidate for inclusion in a study understands how the study is to be conducted could influence the number of individuals inclined to participate in the study. Additionally, the women were interviewed some weeks or months following childbirth, which could have affected their memory of the occurrences and, consequently, their comments.

## Conclusions

Deciding to participate in a clinical trial is not an easy decision, particularly when the individual in question is vulnerable and in a critical situation as is the case of a pregnant woman at high risk of delivering prematurely. The fears and uncertainties regarding the future of the pregnancy will affect her decision on whether or not to participate, as will her previous experiences and her awareness of the actual risks she faces. New clinical trials should take this vulnerability into consideration during the recruitment process, ensuring that the approach taken by the health professional includes clear and sufficient information that will allow the patient to feel confident and at ease to decide whether or not to participate.

The role of the healthcare professional involved in the woman’s prenatal care and in following her up during the clinical trial is crucial in answering any questions she may have regarding the risk to which she is exposed and, moreover, regarding the entire study design and protocol that is being proposed, thus enabling her to make a conscious and safe decision. This doctor-patient communication will play a pivotal role in constructing a bond that will continue throughout the entire study, increasing participant satisfaction and facilitating follow-up.

## Additional file

Additional file 1: Questionnaire about women´s experience of using a treatment to prevent prematurity. (DOCX 26 kb)

## Abbreviations

FDA: United States Food and Drug Administration
ICU: Intensive Care Unit
IMIP: *Instituto de Medicina Integral Professor Fernando Figueira*
SUS: Brazilian National Health Service
UNICAMP: University of Campinas
