# Supplementary material for: Reasons given by pregnant women for participating in a clinical trial aimed at preventing premature delivery: a qualitative analysis
Source: BMC Pregnancy Childbirth. 2019 Mar 20;19:97. doi: 10.1186/s12884-019-2240-8 (PMC6425624; doi:10.1186/s12884-019-2240-8)
Supplement: Supplementary file 1 — Questionnaire about women´s experience of using a treatment to prevent prematurity. (DOCX 26 kb) [file 12884_2019_2240_MOESM1_ESM.docx]

**Experience of pregnant women regarding two preventive treatments strategies for the prevention of preterm birth**

**Questionnaire**

**Age, city, education level, weeks post partum**

**No. Woman P5: |___|___|___| Identification: |___|___|___|**

**INTERVIEWER: _____________________________**

**DATE:___/___/___**

**==============================================================**

**INSTRUCTION 1: INTERVIEWER. SAY: Now I will make some questions about your experience regarding the treatment you did because the high risk you had for preterm birth.**

1. When the doctor explained to you about your high risk for premature birth because you had a short cervix, did you understand all explanation?

[ 1 ]YES [ 2 ] NO [ 3 ] NOT FULLY

**GO TO QUESTION 3**

2. Why?

______________________________________________________________________

______________________________________________________________________

3. Which treatment did you do?:vaginal progesterone or cervical pessary plus vaginal progesterone?

[ 1 ]PROGESTERONE [ 2 ] CERVICAL PESSARY + PROGESTERONE

[ 8 ]DO NOT KNOW/DO NOT REMEMBER**🡪END OF THE INTERVIEW**

4. Did you understand all doctor’s explanations of the proposed treatment?

[ 1 ]YES [ 2 ] NO [ 3 ] NOT FULLY

**GO TO QUESTION 6**

5. Why?

______________________________________________________________________

______________________________________________________________________

6. Did you soon accept this treatment or did you have any doubts before accepting?

[ 1 ]I SO ACCEPTED**→ GO TO QUESTION 10**

[ 2 ]I HAD doubts BEFORE ACCEPTING

7. What were your doubts?

______________________________________________________________________

______________________________________________________________________

8. Were your doubts solved?

[ 1 ]YES [ 2 ] NO [ 3 ] NOT FULLY

**GO TO QUESTION 10**

9. What were your doubts?

______________________________________________________________________

______________________________________________________________________

10. Finally, what made you decide to undergo the proposed treatment?______________________________________________________________________

______________________________________________________________________

11. During the treatment, did you have any problems that you consider were caused by this treatment _______ (NAME OF THE TREATMENT)?

[ 1 ]YES [ 2 ] NO [ 3 ] DO NOT KNOW/DO NOT REMEMBER **GO TO QUESTION 16 GO TO QUESTION 16**

12. What problems did you have?

(CHECK AS MANY ALTERNATIVES ARE NECESSARY) **INSTRUCTION2 -FOR INTERVIEWER: CHECK ALL ALTERNATIVES SPONTANEOUSLY SPOKEN AND THEN ASK HERFOR THE OTHERS ALTERNATIVES.**

|  | **ESPONTANEUS** | **PROVOKED** |
| --- | --- | --- |
| [ 1 ] Vaginal dischargewithout odor |  |  |
| [ 2 ] Vaginal dischargewith odor |  |  |
| [ 3 ] Colic |  |  |
| [ 4 ] Nervousness |  |  |
| [ 5 ] Constipation |  |  |
| [ 6 ] Others. Whatwerethey? _____________ |  |  |

|  |  |
| --- | --- |

13. Did you talk to someone to deal with these problems?

[ 1 ]YES [ 2 ] NO **→ GO TO QUESTION 16**

14. Who did you talk to?(CHECK AS MANY ALTERNATIVES ARE NECESSARY) **INSTRUCTION 3 – FOR INTERVIEWER:CHECK ALL ALTERNATIVES SPONTANEOUSLY SPOKEN AND THEN ASK HER FOR THE OTHERS ALTERNATIVES.**

|  | **ESPONTANEUS** | **PROVOKED** |
| --- | --- | --- |
| [ 1 ] Prenatal health care professional (doctor, nurse) |  |  |
| [ 2 ] P5 health professional |  |  |
| [ 3 ] Husband/companion |  |  |
| [ 4 ] Family |  |  |
| [ 5 ] Friends, neighborS |  |  |
| [ 6 ] Otherperson. Who?__________ |  |  |

|  |  |  |
| --- | --- | --- |

**FILTER1.**SIGN THE CORRECT ALTERNATIVE BY RESPONSE TO QUESTION 14.

[ 1 ] ALTERNATIVE 1 AND/OR 2SIGNEDS

[ 2 ] ALTERNATIVE 1 AND 2 **NOT SIGNEDS** → **GO TO QUESTION 16**

15. What the prenatal health care professional or P5 health care professionals said to you?

______________________________________________________________________

______________________________________________________________________

16. In general, do you think it was easy or difficult to do the treatment?

[ 1 ]EASY🡪 IF WOMAN USES PESSARY 🡪**GO TO QUESTION 18**

**IF WOMAN USES ONLY PROGESTERONE 🡪 GO TO QUESTION 32**

[ 2 ] DIFFICULT

[ 3 ] MORE OR LESS

[ 4 ]I DON´T KNOW 🡪**GO TO QUESTION 18, IF WOMAN USES PESSARY**

- **GO TO QUESTION 32, IF WOMANONLYUSES PROGESTERONE**

17.Why? (YOU CAN MARK MORE THAN ONE OPTION)

[ 1 ]I WAS WORRIED WITH ANY HARM ON MY BABY

[ 2 ]IT WAS UNCOMFORTABLE

[ 3 ]IT WAS DIFFICULT TO INSERT THE PROGESTERONE ON MY VAGINA

[ 4 ] OTHERS. WHAT WERE THEY? ___________________________________

**FILTER 2.**SIGN THE CORRECT ALTERNATIVE BY RESPONSE TO QUESTION3.

[1 ] ALTERNATIVE 1 (VAGINAL PROGESTERONE) SIGNED🡪**GO TO QUESTION A 33**

[ 2 ] ALTERNATIVE 2 (VAGINAL PROGESTERONE + CERVICAL PESSARY) SIGNED

18. During the last pregnancy, did you have colics before cervical pessary insertion?

[ 1 ]YES [ 2 ] NO [ 3 ] DO NOT KNOW/DO NOT REMEMBER

**GO TO QUESTION 20 GO TO QUESTION 20**

19. Was the pain: mild, moderate or severe?

[ 1 ]MILD [ 2 ] MODERATE[ 3 ] SEVERE

20. Did you have colics after the cervical pessary insertion?

[ 1 ] YES [ 2 ] NO [ 3 ] DO NOT KNOW/DO NOT REMEMBER

**GO TO QUESTION 22 GO TO QUESTION 22**

21. Was the pain: mild, moderate or severe?

[ 1 ] MILD [ 2 ] MODERATE [ 3 ] SEVERE

22. Did you have vaginal discharge before cervical pessary insertion?

[ 1 ] YES [ 2 ] NO [ 3 ] DO NOT KNOW/DO NOT REMEMBER

**GO TO QUESTION 24 GO TO QUESTION 24**

23. Was the vaginal discharge: mild, moderate orsevere?

[ 1 ] MILD [ 2 ] MODERATE [ 3 ] SEVERE

24. Did you have vaginal discharge after cervical pessary insertion?

[ 1 ] YES [ 2 ] NO [ 3 ] DO NOT KNOW/DO NOT REMEMBER

**GO TO QUESTION 26 GO TO QUESTION 26**

25. Was the vaginal discharge: mild, moderate or severe?

[ 1 ] MILD [ 2 ] MODERATE [ 3 ] SEVERE

1. Did you fell pain during cervical pessary insertion?

[ 1 ] YES [ 2 ] NO [ 3 ] DO NOT KNOW/DO NOT REMEMBER

**GO TO QUESTION 28 GO TO QUESTION 28**

1. Was the pain: mild, moderate or severe?

[ 1 ] MILD [ 2 ] MODERATE [ 3 ] SEVERE

1. Did you fell pain during removal the cervical pessary?

[ 1 ] YES [ 2 ] NO [ 3 ] DO NOT KNOW/DO NOT REMEMBER

**GO TO QUESTION 30 GO TO QUESTION 30**

1. Was the pain: mild, moderate or severe?

[ 1 ] MILD [ 2 ] MODERATE [ 3 ] SEVERE

30. Did you have vaginal intercourse using cervical pessary?

[ 1 ] YES [ 2 ] NO [ 3 ] DO NOT KNOW/DO NOT REMEMBER

**GO TO QUESTION 32 GO TO QUESTION 32**

31. Did you have any difficulty?

______________________________________________________________________

______________________________________________________________________

32. Did you received any advice to have no vaginal intercourse ?

[ 1 ] YES [ 2 ] NO [ 3 ] DO NOT KNOW/DO NOT REMEMBER

**GO TO QUESTION 34 GO TO QUESTION 34**

33. In a new pregnancy, if it was necessary, would you do this treatment again?

[ 1 ] YES [ 2 ] NO [ 3 ] DO NOT KNOW/DO NOT REMEMBER

34. Would you recommend this treatment to a friend or family with high risk for preterm birth?

[ 1 ] YES [ 2 ] NO [ 3 ] DO NOT KNOW/DO NOT REMEMBER

**GO TO QUESTION 36**

35. Why? (YOU CAN MARK MORE THAN ONE OPTION)

[ 1 ]I WAS WORRIED WITH ANY HARM ON MY BABY

[ 2 ] IT WAS UNCOMFORTABLE

[ 3 ] IT WAS DIFFICULT TO INSERT THE PROGESTERONE ON MY VAGINA

[ 4 ]I COULD NOT HAVE SEXUAL INTERCOURSE

[ 5 ] OTHERS. WHATWERE THEY? ________________________________

______________________________________________________________

36. In your opinion, did the treatment work or not? Why?

______________________________________________________________

______________________________________________________________

**End of the interview**
